# Supplementary material for: H2 formation via non-Born-Oppenheimer hydrogen migration in photoionized ethane
Source: Nat Commun. 2023 Aug 16;14:4951. doi: 10.1038/s41467-023-40628-9 (PMC10432507; doi:10.1038/s41467-023-40628-9)
Supplement: Supplementary file 1 — Supplementary Information [file 41467_2023_40628_MOESM1_ESM.pdf]

Supplementary Information for:
H_2 Formation via Non-Born-Oppenheimer Hydrogen Migration in Photoionized Ethane

Yizhang Yang[1*], Hao Ren[2*], Ming Zhang[3*], Shengpeng Zhou[1], Xiangxu Mu[3], Xiaokai Li[1], Zhenzhen Wang[1], Ke Deng[1], Mingxuan Li[1], Pan Ma[1], Zheng Li[3,4,5,†] Xiaolei Hao[2,‡] Weidong Li[6], Jing Chen[6,7], Chuncheng Wang[1,§] and Dajun Ding[1¶]

[1]*Institute of Atomic and Molecular Physics and Jilin Provincial Key Laboratory of Applied Atomic and Molecular Spectroscopy, Jilin University, Changchun 130012, China*

[2]*Institute of Theoretical Physics and Department of Physics, State Key Laboratory of Quantum Optics and Quantum Optics Devices, Collaborative Innovation Center of Extreme Optics, Shanxi University, Taiyuan 030006, China*

[3]*State Key Laboratory for Mesoscopic Physics and Frontiers Science Center for Nano-Optoelectronics, School of Physics, Peking University, Beijing 100871, China*

[4]*Collaborative Innovation Center of Extreme Optics, Shanxi University, Taiyuan, Shanxi 030006, China*

[5]*Peking University Yangtze Delta Institute of Optoelectronics, Nantong, Jiangsu 226010, China*

[6]*Shenzhen Key Laboratory of Ultraintense Laser and Advanced Material Technology, Center for Advanced Material Diagnostic Technology, and College of Engineering Physics, Shenzhen Technology University, Shenzhen 518118, China and*

[7]*Institute of Applied Physics and Computational Mathematics, P. O. Box 8009, Beijing 100088, China*

(Dated: August 10, 2023)

Supplementary note 1: Experimental Setup

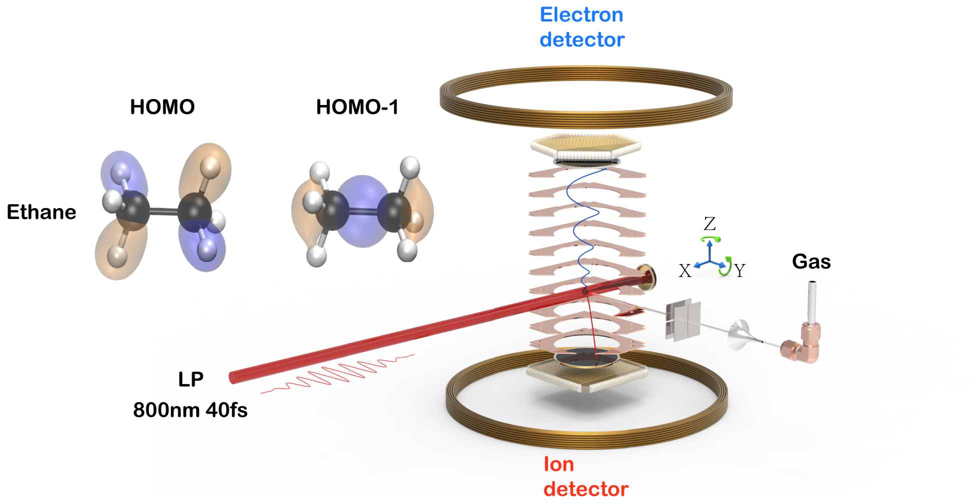

Supplementary Figure 1: Schematic of the COLTRIMS setup. The ethane molecules were fed into the reaction chamber supersonically via the skimmer and slit, and then ionized by the linearly polarized (LP) laser pulses. The produced photoelectrons and ions were detected by the microchannel plates (MCP) detector and delay-line detector, respectively. The isosurfaces of the e_g (HOMO) and a_{1g} (HOMO-1) orbitals of ethane are displayed, with an isovalue of $0.1\text{Å}^{-3/2}$ (orange) and $-0.1\text{Å}^{-3/2}$ (blue).

The experimental setup is presented in Supplementary Figure 1. The linearly polarized laser pulses centered at a wavelength of 800 nm and with a pulse duration of 40 fs, were generated using a Ti:Sapphire laser operating at a

---

* These authors contribute equally to this work.

repetition rate of 1 kHz. The laser beam was focused onto the molecular beam using a mirror with a focal length of 75 mm and its polarization was oriented vertically with respect to the time-of-flight (TOF) direction. The process of molecular ionization and fragmentation induced by the laser pulses was investigated by the cold target recoil ion momentum spectroscopy (COLTRIMS). The produced electrons and ions were detected in coincidence by two time- and position-sensitive detectors in a uniform magnetic field and electric field configuration.

Supplementary note 2: Surface hopping molecular dynamics simulation and frequency analysis

In order to validate the interpretation of the non-adiabatic effect in the experiment, we performed fewest-switches surface hopping (FSSH) molecular dynamics (MD) simulation on the CASSCF(5,5)//6-31G* level to determine the branching ratio of the dissociative H_2 formation channel. The MD simulation includes non-adiabatic couplings for the lowest three doublet cationic states in the adiabatic representation, denoted as D_0, D_1 and D_2, which are correlated with the cationic ^2E_g and $^2A_{1g}$ states in the $D_{3\mathrm{d}}$ symmetric geometry. The D_0 state is correlated with the diabatic $^2A_{1g}$ state in the dissociation limit, and the energy barrier for H_2 formation reaction initiated from Franck-Condon geometry $C_2H_6^+$ is 0.68 eV. As shown in Figure 4 of the main text, the FSSH simulation results in a 67% branching ratio of the dissociative H_2 formation channel in the ^2E_g state, and the calculated branching ratio of the bound parent ion channel in the $^2A_{1g}$ state is 31%, showing consistency with the experimental results. The FSSH molecular dynamics simulation results indicate that the non-adiabatic coupling between ^2E_g and $^2A_{1g}$ states is crucial for the H_2 formation dynamics of ethane cation $C_2H_6^+$.

Furthermore, we performed frequency analysis for the MD trajectories, and investigate its relationship with the frequency distribution for the CI geometries with H_2 formation character. The result, together with the frequency analysis of the experimentally measured $C_2H_4^{2+}$ + H_2 yields oscillation, are shown in Figure 5 in the main text. Continuous wavelet transformation (CWT) method are used to obtain the time and frequency distribution $I_{\mathrm{CWT}}(\omega, t)$ from velocity autocorrelation function

$$f(t) = \left\langle \sum_i \vec{v}_i(t) \cdot \vec{v}_i(0) \right\rangle , \tag{1}$$

where i is index of atoms, and $\langle \cdots \rangle$ represents the average over molecular dynamics trajectories. The CI geometry shown in Fig. 1c in the main text is optimized by the quantum chemistry packages Molpro [1] initialized from the transition state geometry of the H_2 formation reaction $C_2H_6^+ \to C_2H_4^+ + H_2$. We define the characteristic frequency distribution of CI geometry to be

$$I_{\mathrm{CI}}(\omega) = \sum_i \delta(\omega - \omega_i) , \tag{2}$$

where ω_i is the i-th vibrational mode frequency of the optimized geometry around the conical intersection, and the vibrational modes are shown in Supplementary Figure 2. The peak time for the overlap between $I_{\mathrm{CWT}}(\omega, t)$ and $I_{\mathrm{CI}}(\omega)$ corresponds to the characteristic time when $C_2H_6^+$ reaches the CI geometry.

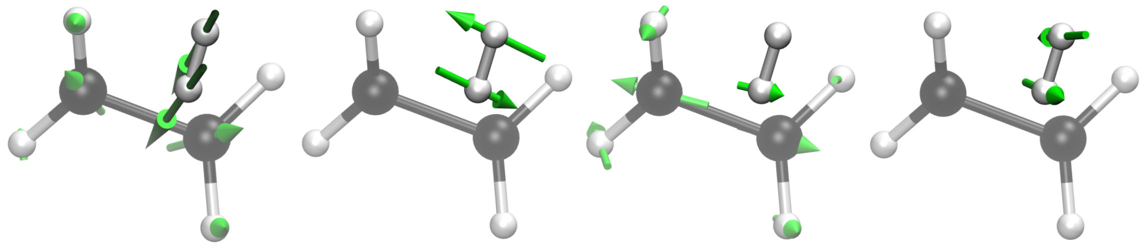

Supplementary Figure 2: Mass weighted vibrational vectors for the vibrational modes of the optimized geometry around the representative CI geometry. Only the vibrational modes assigned in Fig. 5b of the main text are shown. The vibrational frequencies are 114 cm^{-1}, 141 cm^{-1}, 156 cm^{-1}, and 202 cm^{-1}, respectively.

Supplementary note 3: Fitting procedure details for above-threshold ionization spectrum

The above-threshold ionization (ATI) spectroscopy contains typical comb structures, which are spaced by the photon energy of the laser pulses. These combs arise from electrons tunneling from the two highest occupied molecular orbitals (HOMO and HOMO-1) and their energy offsets reflect the differences in ionization potentials between these orbitals. Consequently, the comb structures observed in the ATI spectra can serve as a signature to disentangle contributions from multiple orbitals in ATI spectroscopy. In the low-intensity region, polyatomic molecules, including ethylene and other molecules mentioned in the literature [2], exhibit high contrast ATI combs. For ethane, the non-adiabatic coupling between the ionic ground and excited states plays a key role in the formation of H_2, as depicted in Figure 1 of the main text. The ATI combs in coincidence with the parent ion and fragment consist of two components corresponding to the cationic ground state ^2E_g (resulting from HOMO ionization) and the excited state $^2A_{1g}$ (resulting from HOMO-1 ionization), respectively. Considering the present peak laser intensity, direct tunneling ionization (TI) from HOMO-2 can be neglected, as the ionization rate for HOMO-2 is two orders of magnitude smaller than that for HOMO, according to molecular Ammosov-Delone-Krainov (ADK) calculations. To disentangle the contributions from the ^2E_g and $^2A_{1g}$ electronic states, we applied a global fitting procedure to reconstruct the observed ATI spectra from different channels, as shown in Supplementary Figure 3. To minimize the influence of the Freeman resonance (FR), the fitting procedure ignores the spectrum below 1 eV. Additionally, extending the energy range beyond 7 eV does not affect the fitting results, as the ATI combs are completely smeared out. Thus, the fitting is performed within the energy range of 1∼7 eV in the ATI spectrum.

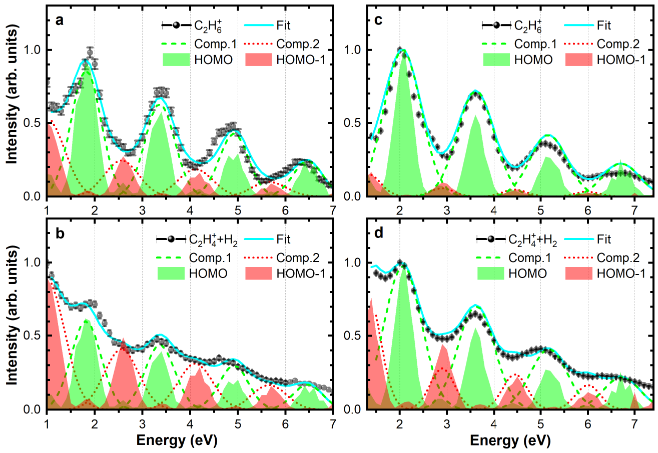

Supplementary Figure 3: The measured ATI spectra in coincidence with the parent ion channel (a)(c) and H_2 formation channel (b)(d) of ethane cation. In panels (a) and (b), the laser intensity is 88 TW/cm^2, while in panels (c) and (d), the laser intensity is 82 TW/cm^2. All the spectra are fitted with two series of Gaussian functions (see Supplementary Equations 3-5). The solid cyan line represents the fitted curve, which reproduced the measurement (black) very well. The fitted components, denoted as Comp. 1 and Comp. 2, correspond to the contributions from HOMO and HOMO-1 ionization, respectively. The calculated ATI spectras obtained using the Coulomb-corrected strong-field approximation (CCSFA) approach are displayed in the green and red shadows, which corresponds to the ionization from HOMO and HOMO-1 orbitals of ethane, respectively.

The fitting function we used is as follows:

$$\mathrm{Y}_j(x) = B_j\% \times (\mathrm{HOMO}) + (1 - B_j\%) \times (\mathrm{HOMO} - 1)\,, \quad j = 1, 2\,, \tag{3}$$

$$\mathrm{HOMO} = \sum_{i=1}^{4} A_{1i} \times G(x, [\mu_1 + k \times (i-1), \sigma_1^2])\,, \tag{4}$$

$$\mathrm{HOMO} - 1 = \sum_{i=1}^{4} A_{2i} \times G(x, [\mu_2 + k \times (i-1), \sigma_2^2])\,, \tag{5}$$

where Y_j are the measured ATI spectra signals in coincidence with the parent ion ($j = 1$) and fragment ion ($j = 2$) of ethane, which are normalized with respect to the total electron counts. $B_j\%$ is the ratio of the ^2E_g state in the

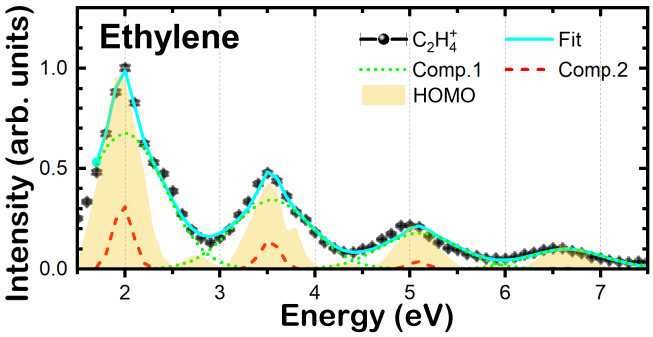

Supplementary Figure 4: The ATI spectra in coincidence with the parent ion of ethylene with a laser intensity of 76 TW/cm^2. The solid cyan line represents the fitted curves which reproduce the measurement (black) very well. Comp. 1 and Comp. 2 represent the two fitted components. The calculated ATI spectrum from HOMO with CCSFA is shown as the yellow shadow.

Intensity	88 TW/cm^2		82 TW/cm^2	
Parameter	Fitting	Error	Fitting	Error
$\mu_1 - \mu_2$	0.80 eV	0.13 eV	0.72 eV	0.15 eV
B_1%	69%	10%	93%	15%
B_2%	48%	11%	75%	16%

Supplementary Table 1: Results of the fitting parameters from ATI spectrum. $\mu_1 - \mu_2$ represents the energy difference between the two fitted energy combs. B_1% is the contribution corresponding to ionization from HOMO electron orbital in the parent ion channel, and B_2% is that for H_2 formation channel.

Y_j spectrum, $A \times G(x, [\mu, \sigma^2])$ denotes the Gaussian function with amplitude A, center energy μ and energy width of standard deviation σ, k is the photon energy (1.55 eV). Supplementary Equations 4 and 5 are the sum of four Gaussian distributions, which represent the energy combs ionized from the HOMO and HOMO-1, respectively. The full width at half maximum (FWHM) of each component is a fitted parameter that takes into account the laser intensity volume effect. The energy offset between the two fitted ATI combs is determined to be 0.80 (±0.13) eV. The detailed results are shown in Supplementary Table 1. For the 88 TW/cm^2 laser intensity case, the fitted amplitudes for Comp. 1, which correspond to the contributions from the ^2E_g state, are 69(±10)% and 48(±11)% for the parent ion channel and H_2 formation channel, respectively. To estimate the influences of background signals on the fitting procedure, the events from false coincidence and noise in the raw ATI spectra need to be considered. In our measurement, the count rates for electrons and ions are approximately 0.1/pulse and 0.075/pulse, respectively, with a total detection efficiency of around 50%. Therefore, the estimated false coincidence rate is 3%. The average noise level in each measurement is below 5%. To determine the total error of the fitting results, we added a random noise level of 10% to the total counts of each energy bin in the raw ATI spectrum and generated 1000 sampled spectra. The same fitting procedure was then applied to these spectras, and the error of the fitting results was obtained and shown in Supplementary Table 1 for two laser intensities. We utilized ethylene as a benchmark for the analysis of ATI spectra. Ethylene exhibits an energy gap of approximately 2 eV between its first excited and ground ionic states. In the fitting process, we considered two components and observed two overlapping energy combs from the fitting, as shown in Supplementary Figure 4, suggesting that ionization from the HOMO is dominant in ethylene.

Supplementary note 4: Reconstruction of PMDs corresponding to ^2E_g and $^2A_{1g}$ states

The measured and calculated PMDs of ethylene are shown in Supplementary Figure 5c and d, respectively, demonstrating good agreement and proving the validity of CCSFA calculation. Figure 5e and f show the measured raw PMDs in coincidence with the parent ion and H_2 formation channels for ethane, while the calculated PMDs for the HOMO (^2E_g state) and HOMO-1 ($^2A_{1g}$ state) ionization are presented in Figure 5h and j, respectively. A discrepancy between the PMDs from the raw measurement and the calculations can be observed. The distinct angular distributions cannot be easily interpreted using the simple explanation commonly applied to diatomic molecules [3–5]. Based on our molecular dynamics (MD) simulations and fitting approach, we suggest that both the ^2E_g and $^2A_{1g}$ states

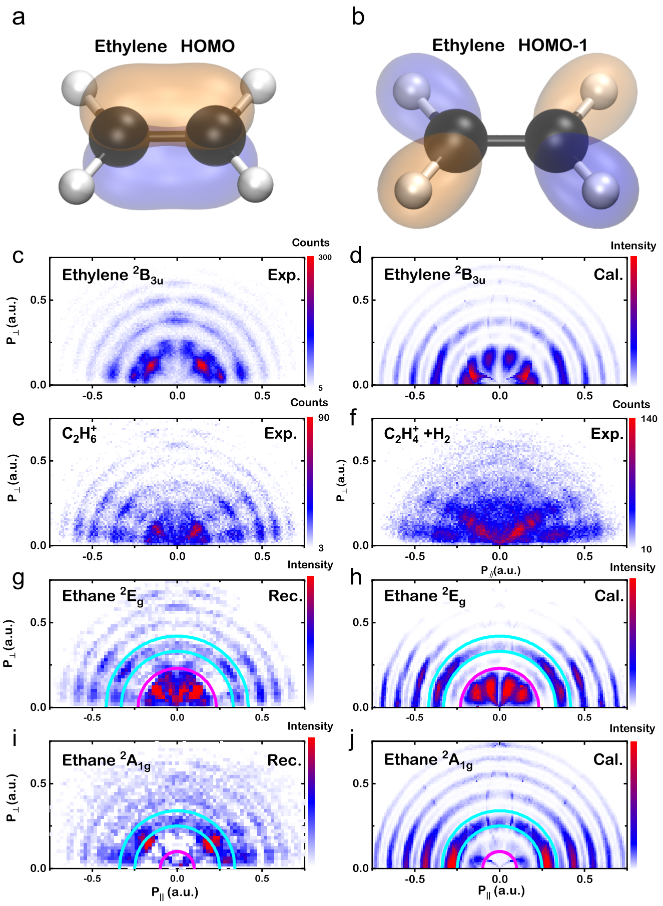

Supplementary Figure 5: The reconstructed PMDs compared with the raw experimental datas and theoretical simulations. a, b The isosurfaces of HOMO and HOMO-1 of ethylene with an isovalue of $0.1Å^{-3/2}$ (orange) and $-0.1Å^{-3/2}$ (blue), respectively. **c, d** The measured and calculated PMDs of ethylene respectively. **e, f** The measured PMDs in coincidence with the parent ion and H_2 formation channels respectively. **g, h** The reconstructed PMDs corresponding to the ^2E_g and $^2A_{1g}$ states of ethane respectively. **i, j** The calculations compared to the PMDs in (g) and (h). The laser intensity is 76 TW/cm^2 for ethylene while 88 TW/cm^2 for ethane. In (g) and (h), the fanning out stripes are located within the pink semicircle for the ^2E_g state. In (i) and (j), the arm-like structure is dominant within the pink semicircle. The jet-like structures in the ATI spectra are emphasized by the two solid blue curves.

contribute to the PMDs of the parent ion and H_2 formation channels due to non-adiabatic coupling dynamics through conical intersections (CIs). According to the fitted values of $B_1\%$ and $B_2\%$, the ATI spectrum of ionization from the HOMO and HOMO-1 can be reconstructed, as discussed in Supplementary note 3. It shows the measured PMDs of the parent ion and H_2 formation channels are linear combinations of PMDs corresponding to ^2E_g (HOMO) and $^2A_{1g}$(HOMO-1) states with different relative ratios, which can be described as following:

$$\mathrm{PMD}_{i,j}[x,y] \quad = \quad B_{i,j}\% \times (\mathrm{HOMO_j}[x,y]) + (1 - B_{i,j}\%) \times ((\mathrm{HOMO}-1)_{i,j}[x,y])\,, \quad i = 1,2, \quad j = 1,2,..,6\,. \quad (6)$$

Thus the PMD corresponding to HOMO and HOMO-1 can be reconstructed from the measured PMDs once we know $B_j\%$. However, directly applying the same method used for ATI to reconstruct the PMD solely from the HOMO or HOMO-1 is not feasible, as the angular-dependent TI yields have different relative contributions of the two components for various electron emission angles. To overcome this challenge, we divided the PMD into six angular regions and obtained the relative yields of the two components by fitting each integrated ATI spectra (formalized as Supplementary Equation 6). The value of six is chosen simply because of the limit of the statistics of electron counts. Since the PMDs of the parent ion and H_2 formation channels both contain contributions from the ^2E_g and $^2A_{1g}$ states with different relative ratios, the PMD exclusively from the ^2E_g state can be reconstructed by subtracting the normalized PMD of H_2 formation channel from that of parent ion channel, where the normalization ratio is determined by $B_1\%$.

Intensity	88 TW/cm^2						82 TW/cm^2					
Angle	0°-30°	30°-60°	60°-90°	90°-120°	120°-150°	150°-180°	0°-30°	30°-60°	60°-90°	90°-120°	120°-150°	150°-180°
$\mu_1 - \mu_2$	0.80eV	0.88eV	0.82eV	0.88eV	0.80eV	0.9eV	0.73eV	0.70eV	0.75eV	0.73eV	0.70eV	0.67eV
B_1%	86%	70%	70%	55%	76%	68%	90%	90%	85%	100%	100%	90%
B_2%	55%	42%	47%	31%	52%	45%	60%	75%	65%	70%	80%	50%

Supplementary Table 2: Angular resolved fitting results for the reconstruction of the PMD from ^2E_g state to the parent ion channel and the H_2 formation channel. Definition of the parameters $\mu_1 - \mu_2$, B_1% and B_2% are the same as in Supplementary Table 1.

Similarly, we obtained the PMD of the $^2A_{1g}$ state.

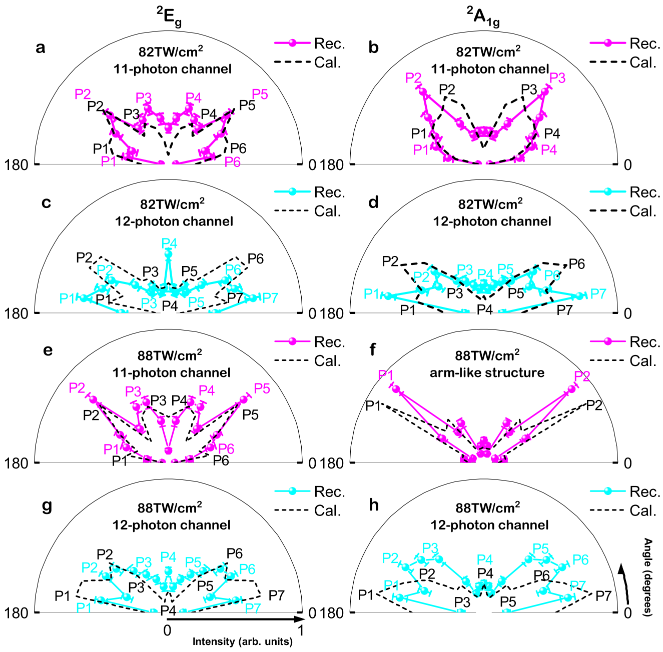

Supplementary Figure 6: The integrated angular distributions for the low-energy structure in the PMD and the ATI peak for the ^2E_g and $^2A_{1g}$ states of ethane for two peak laser intensities. The experimentally reconstructed angular distributions and the calculations are shown as the solid and dashed lines, respectively. (a)(b)(e)(f) show the distributions of the fanning out stripes and arm-like structure, while (c)(d)(g)(h) show the angular distributions of ATI peak (12-photon channel) for ^2E_g and $^2A_{1g}$ states. The numbers of the maxima are labeled next to the position of the peaks. All the curves have been symmetrized.

The reconstructed PMDs of the ^2E_g and $^2A_{1g}$ states, based on the fitted values (Supplementary Table 2), are shown in Supplementary Figure 5g and i. This method can also be applied to the case of 82 TW/cm^2 laser intensity, enabling the reconstruction of the PMDs shown in Fig. 3c and d in the main text. These reconstructed PMDs demonstrate good agreement with the calculations based on the CCSFA approach. The characteristic features, such as the jet-like distributions of the ATI ring (within the solid blue curves) and the low-energy structures (within the pink semicircle), are well reproduced by the calculations, validating the accuracy of the reconstructed PMDs (in Supplementary Figure 6). In the case of the ^2E_g state, the fanning out structure contains of six stripes (11-photon channel), and the jet-like structure within ATI ring (12-photon channel) shows seven nodes, which are consistent with the calculations. For the $^2A_{1g}$ state, the jet-like structure within the ATI (12-photon channel) contains seven nodes, matching the calculation. At a higher laser intensity of 88 TW/cm^2, the arm-like structure becomes less pronounced compared to 82 TW/cm^2, mainly due to the electron energy from the 11-photon channel in ATI being close to 0 eV. The quantitative agreement between the extracted and simulated PMDs strongly confirms the reliability of the fitting procedure.

Supplementary note 5: Coulomb-corrected strong-field approximation (CCSFA) approach for molecule ionization.

The transition amplitude $M_{\mathbf{p}}$ in strong-field approximation (SFA) in the form of saddle point approximation can be written in the atomic units as

$$M_{\mathbf{p}} = \sum_s \langle \mathbf{p}+\mathbf{A}(t_{\mathrm{s}}) | \mathbf{r}\cdot\mathbf{E}(t_{\mathrm{s}}) | \psi_0 \rangle \sqrt{\frac{2\pi i}{\partial^2 S/(\partial t_{\mathrm{s}})^2}} \exp[iS(t_{\mathrm{s}})] , \tag{7}$$

where $\mathbf{A}$ and $\mathbf{E}$ are the vector potential and electric field of laser, $|\psi_0\rangle$ is the initial bound state of the electron, $|\mathbf{p}+\mathbf{A}(t)\rangle$ is the Volkov state, $S(t) = \int_{-\infty}^{t} \mathrm{d}t' [\mathbf{p}+\mathbf{A}(t')]^2/2 + I_{\mathrm{p}} t$ is the action. Saddle point solutions t_{s} can be obtained by solving the saddle point equation

$$\left.\frac{\partial S(t)}{\partial t}\right|_{t_{\mathrm{s}}} = 0 \Leftrightarrow \frac{1}{2}[\mathbf{p}+\mathbf{A}(t_{\mathrm{s}})]^2 = -I_{\mathrm{p}}. \tag{8}$$

The above expressions are known as the SFA, which states that once the electron is ejected, Coulomb interaction between the electron and the residual ion is ignored.

The CCSFA theory adds corrections to the SFA by considering the effect of Coulomb field to the trajectories and action of ionized electrons [6]

$$M_{\widetilde{\mathbf{p}}} = \sum_s \langle \mathbf{p}+\mathbf{A}(t_{\mathrm{s}}) | \mathbf{r}\cdot\mathbf{E}(t_{\mathrm{s}}) | \psi_0 \rangle \sqrt{\frac{2\pi i}{\partial^2 S/(\partial t_{\mathrm{s}})^2}} \exp[iS'(t_{\mathrm{s}})] . \tag{9}$$

Here $\widetilde{\mathbf{p}}$ is the asymptotic momentum disturbed by the Coulomb field, $S'(t_{\mathrm{s}})$ is the corrected action which reads

$$S'(t_{\mathrm{s}}) = -\int_{t_{\mathrm{s}}}^{t_r} \left\{ \frac{[\mathbf{p}+\mathbf{A}(\tau)]^2}{2} - \frac{1}{\sqrt{\mathbf{r}^2(\tau)}} \right\} d\tau - \int_{t_r}^{T_p} \left[\frac{\widetilde{\mathbf{v}}^2(\tau)}{2} + V(\mathbf{r}) \right] d\tau + I_p (t_{\mathrm{s}} - T_p) . \tag{10}$$

The first integral in the right hand of Supplementary Equation 10 denotes the sub-barrier action, where $\mathbf{p}$ is the undisturbed asymptotic momentum, the upper limit t_r is the real part of t_{s}. The second integral denotes the action obtained after the electron leaving the tunnel exit. The instantaneous velocity $\widetilde{\mathbf{v}}(t)$ is calculated by Newton equation $d\widetilde{\mathbf{v}}(t)/dt = \mathbf{E}(t) - \partial V(\mathbf{r})/\partial \mathbf{r}$ with $V(r) = -1/r$. The electrons propagate in the laser field until the pulse ends at time T_p, then the final asymptotic momentum $\widetilde{\mathbf{p}}$ can be analytically obtained using the Kepler's law. Finally, the trajectories are summed coherently to obtain different kinds of distributions.

The initial state of molecules $|\psi_0(t)\rangle$, within the fixed-nuclei approximation, can be written as a linear combination of atomic orbitals (LCAO). Here we consider the symmetry properties of molecular orbitals under the mirror reflection perpendicular to C-C bond. The C_2H_4 molecule has $D_{2\mathrm{h}}$ symmetry and the C_2H_6 molecule has $D_{3\mathrm{d}}$ symmetry, and their HOMO and HOMO-1 orbital symmetry properties are shown in Supplementary Figure 5(a,b) and Figure 1, respectively. The resulting LCAO orbitals can be written as combinations of atomic orbital pairs. In each pair, the two centers of the atomic orbitals are symmetric about the origin just as in a homonuclear diatomic molecule, such that pairs of atomic orbitals form symmetric ($\gamma = 1$) and antisymmetric ($\gamma = -1$) combinations:

$$\psi_0(\mathbf{r}) = \sum_a c_a \left[\psi_a^{(0)}(\mathbf{r} + \mathbf{R}_a/2) + \gamma \psi_a^{(0)}(\mathbf{r} - \mathbf{R}_a/2) \right] , \tag{11}$$

where $\mathbf{R}_a$ denotes the relative nuclear coordinates, the subscript a denotes different atom pairs and γ can be 1 or -1 depending on the symmetry. We show the contributions from the different centers of the molecules to the molecular orbitals in Supplementary Table 3.

After substituting the wavefunction Supplementary Equation 11 into Equation 9 according to the dressed modified molecular SFA, the transition amplitudes can be rewritten as

$$M_{\widetilde{\mathbf{p}}} = \sum_{s,a} c_a f_{\gamma,a}(\mathbf{p}, \mathbf{R}_a) \langle \mathbf{p}+\mathbf{A}(t_{\mathrm{s}}) | \mathbf{r}\cdot\mathbf{E}(t_{\mathrm{s}}) | \psi_a^{(0)} \rangle \sqrt{\frac{2\pi i}{\partial^2 S/(\partial t_{\mathrm{s}})^2}} \exp[iS'(t_{\mathrm{s}})] , \tag{12}$$

	C_2H_4	C_2H_6
HOMO	(C$p\pi$+C$p\pi$)	(C$p\pi$-C$p\pi$) +(H+H)$_1$-(H+H)$_2$
HOMO-1	(C$p\sigma$-C$p\sigma$) -(H+H)$_1$+(H+H)$_2$	(C$p\sigma$-C$p\sigma$) -(H+H)$_1$-(H+H)$_2$-(H+H)$_3$

Supplementary Table 3: Composition of molecular orbitals.

I_p(eV)	C_2H_4	C_2H_6
HOMO	10.5 b_{3u}	11.5 e_g
HOMO-1	12.5 b_{3g}	12.2 a_{1g}

Supplementary Table 4: Ionization potentials and symmetries of molecular orbitals.

where

$$f_{\gamma,a}\left(\mathbf{p},\mathbf{R}_a\right) = \begin{cases} 2\cos\left(\mathbf{p}\cdot\mathbf{R}_a/2\right) & \gamma = 1 \\ 2i\sin\left(\mathbf{p}\cdot\mathbf{R}_a/2\right) & \gamma = -1 \end{cases}, \tag{13}$$

is the interference term between two atomic centers in one atom pair.

For some molecules, not only the HOMO but also the lower-lying orbitals can substantially contribute to the ionization process. Hence, we include both the HOMO and HOMO-1 orbitals in our calculations. In Supplementary Table 4, we show the ionization potentials of different orbitals. The difference in ionization potential between the HOMO and HOMO-1 orbitals for ethylene is 2 eV, indicating a dominant contribution of HOMO to the ionization. Moreover, our calculations reveal that the transition amplitude of HOMO-1 is at least two orders of magnitude smaller than that of HOMO for ethylene. On the other hand, for ethane, the difference in ionization potential between the HOMO and HOMO-1 orbitals is only 0.7 eV. In this case, the contributions from both the HOMO-1 and HOMO orbitals become comparable.

In Supplementary Figure 5e to h, the prominent orbital-dependent features are observed in the low-energy region within the pink semicircle for the $^2A_{1g}$ state, specifically the fanning out structure for the ^2E_g state and the arm-like structure for the $^2A_{1g}$ state. These orbital-dependent structures can be understood based on the parity of the molecular orbitals' wave functions. Within the fixed-nuclei approximation, the numerically calculated molecular initial state for ethane can be given as a linear combination of atomic orbitals (LCAO) for one carbon-carbon (C-C) pair and three hydrogen-hydrogen (H-H) pairs. As shown in Supplementary Figure 7, if we separate the contributions of each atomic pair, we can see that the arm-like structure is prominent in the PMDs of the H-H pairs but absent in the PMD of the C-C pair. The parity of the total wave function, and hence the corresponding PMD, depends on the combination of different atomic pairs. For the ^2E_g state, only two pairs of H-H contribute, and they are combined in odd parity (see Supplementary Table 3). As a result, the contributions of the two H-H pairs to the ionization process cancel each other out, and the total PMD is mainly determined by the contribution of the C-C pair. This is confirmed by the calculated PMD from the C-C pair (Supplementary Figure 7a and e), which reproduces the reconstructed PMD of the ^2E_g state well. However, for the $^2A_{1g}$ state, the three pairs of hydrogen atoms are combined in even parity. Therefore, the total PMD is determined by both the C-C pair and the H-H pairs, resulting in the appearance of the arm-like structure in the very low-energy region.

The strong-field ionization depends on the alignment of molecules. Actually, the simulated energy spectra and PMDs presented in the manuscript are the averaged results among different orientations to mimic the experimental conditions in which the molecules are presumably randomly aligned. In Supplementary Figures 8 and 9, the simulated ionization yields and PMDs for the ^2E_g and $^2A_{1g}$ states of ethane are shown for the various angles between the C-C axis and the laser polarization. According to the calculations, the ionization yield of the ^2E_g state exhibits a strong dependence on the molecular orientation. It reaches a maximum at an angle of 45 degrees between the C-C axis and the laser polarization, while minima are observed at 0 and 90 degrees. Therefore, the orientation-averaged results, as presented in the manuscript, are predominantly determined by the ionization of molecules oriented around 45 degrees. In contrast, the ionization yield of the $^2A_{1g}$ state shows a much weaker dependence on the orientation angle. The different behaviors of the two stated root in the different constitutions of their wave functions. The ionization of the ^2E_g state is mainly influenced by the C-C atomic pair, making it sensitive to molecular orientation. But for the $^2A_{1g}$ state, both C-C pair and H-H pairs contribute to the ionization. The three H-H pairs orienting in different directions smear out the orientation effect.

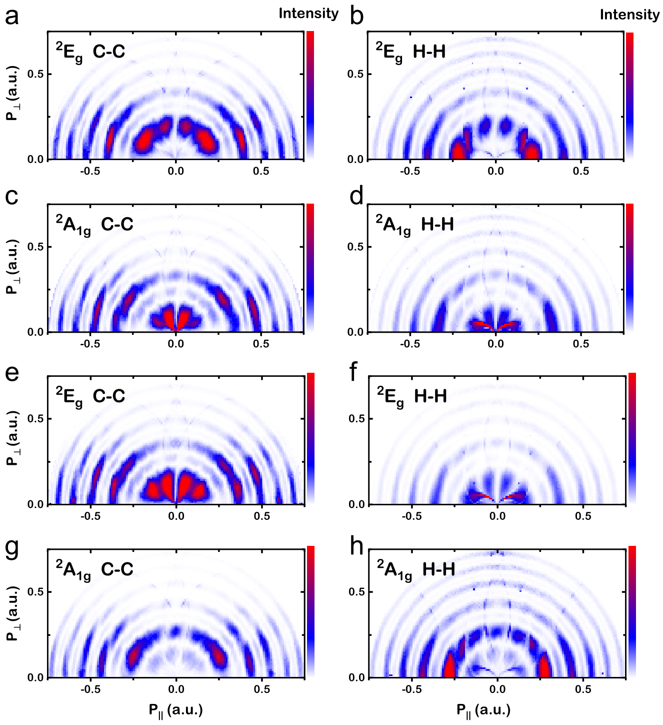

Supplementary Figure 7: The calculated PMDs of two states from the carbon-carbon atom pair and hydrogen-hydrogen pair. The PMDs of ${}^2E_g$ state from these two pairs are shown in (a)(e) and (b)(f). Similarly, the PMD of ${}^2A_{1g}$ state from them are presented in (c)(g) and (d)(h). The laser intensities are 82 TW/cm^2 in (a)-(d) and 88 TW/cm^2 in (e)-(h).

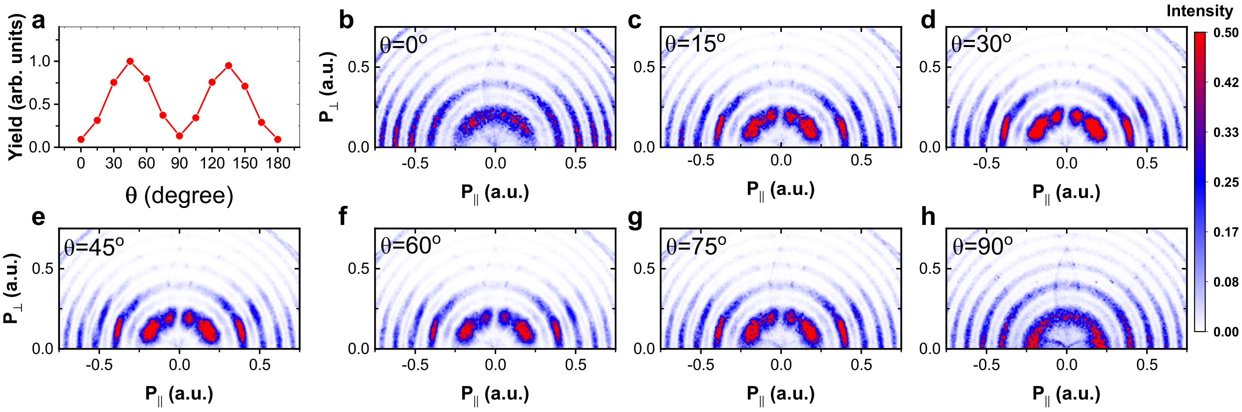

Supplementary Figure 8: The calculated ionization yield (panel a) and photoelectron momentum distributions (PMDs) for the ${}^2E_g$ state of ethane vs the angle θ between the C-C axis (panels b-h). The laser polarization are presented in (a)-(h). The values of θ are inserted in panel b to h.

In addition, the pattern of the PMDs for both states exhibits a weak dependence on molecular orientation. The critical features used to distinguish the two states, i.e., the fanning out structure for the ${}^2E_g$ state and the arm-like structure for the ${}^2A_{1g}$ state, are prominent at nearly all orientation angles. This is because the pattern of the PMD at low energy is primarily determined by the symmetry of the molecular orbital, which is retained at different orientations. Based on these calculations, it is expected that the molecular alignment has a minor effect on the reconstructed results of the two states under the considered experimental conditions.

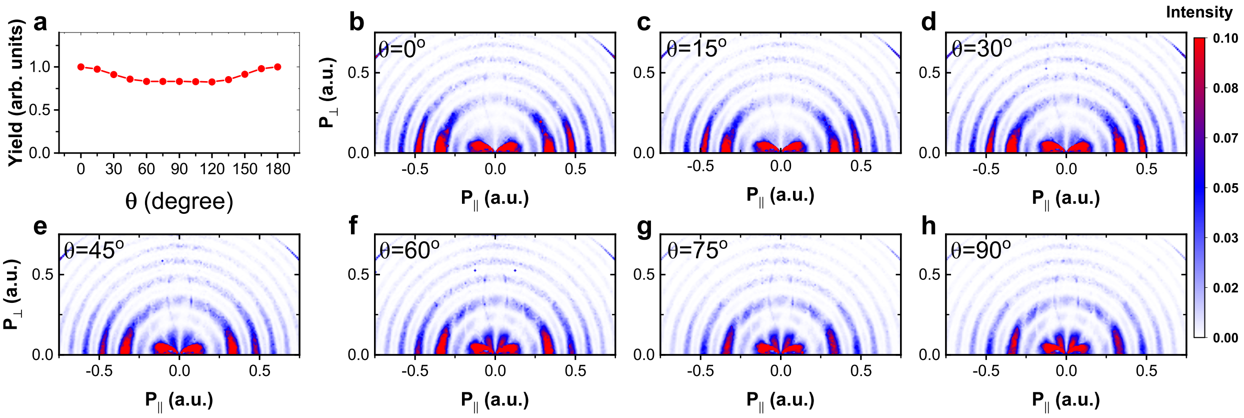

Supplementary Figure 9: The calculated ionization yield (panel a) and photoelectron momentum distributions (PMDs) for the $^2A_{1g}$ state of ethane vs the angle θ between the C-C axis (panels b-h). The values of θ are inserted in panels b-h.

Supplementary note 6: The non-adiabatic coupling dynamics driven by tunneling ionization and Freeman resonance ionization

88 TW/cm^2	Counts	HOMO (Branching ratio)	HOMO-1 (Branching ratio)
Parent	$N_{\rm P}$=22300	$N_{\rm P}\times$69%=15387 (15387/N_E=36($\mp$8)%)	$N_{\rm P}\times$31%=6913 (6913/N_A=19($\pm$9)%)
Dissociation	$N_{\rm D}$=58041	$N_{\rm D}\times$48%=27860 (27860/N_E=64($\pm$8)%)	$N_{\rm D}\times$52%=30181 (30181/N_A=81($\mp$9)%)
Total	$N_{\rm P}$+$N_{\rm D}$=80341	N_E=43247 (N_E/($N_{\rm P}$+$N_{\rm D}$)=54($\mp$11)%)	N_A=37094 (N_A/($N_{\rm P}$+$N_{\rm D}$)=46($\pm$11)%)

82 TW/cm^2	Counts	HOMO (Branching ratio)	HOMO-1 (Branching ratio)
Parent	$N_{\rm P}$=873079	$N_{\rm P}\times$93%=820694 (820694/N_E=33($\mp$7)%)	$N_{\rm P}\times$7%=52385 (52385/N_A=9($\pm$21)%)
Dissociation	$N_{\rm D}$=2267530	$N_{\rm D}\times$75%=1700648 (1700648/N_E=67($\pm$7)%)	$N_{\rm D}\times$25%=566882 (566882/N_A=91($\mp$21)%)
Total	$N_{\rm P}$+$N_{\rm D}$=3140609	N_E=2521342 (N_E/($N_{\rm P}$+$N_{\rm D}$)=80($\mp$14)%)	N_A=619267 (N_A/($N_{\rm P}$+$N_{\rm D}$)=20($\pm$14)%)

Supplementary Table 5: Branching ratios of H_2 formation and parent ion channels for two electronic states obtained from Tunneling ionization. $N_{\rm D}$ and $N_{\rm P}$ stand for the measured total electron counts in coincidence with H_2 formation and parent ion channels. N_E and N_A stand for the derived initial total counts for the ionic ground and excited states. The branching ratio of H_2 formation channel from ionic ground state can be derived by $N_{\rm D}\times B_2\%/N_E$, where $B_2\%$ comes from the fit in Supplementary Table 1. Similar formula is used in the case of parent ion channel.

We established that the non-adiabatically coupled ^2E_g and $^2A_{1g}$ cation states can both contribute to the parent ion and H_2 formation channels. In Supplementary Table 5, we provided the method to extract the branching ratios and listed the important numbers of electron events for two laser intensities. Moreover, we qualitatively show that the sub-cycle TI yield ratios of the HOMO-1 orbital ($N_A/(N_{\rm P}$+$N_{\rm D}$) has an enhancement with increasing the laser intensity from 82 TW/cm^2 to 88 TW/cm^2. These values are shown in the last row of Supplementary Table 5. The error bars of all branching ratios from TI in Figure 4 of the main text are obtained by propagating the error of $B_1\%$ and $B_2\%$ shown in Supplementary Table 1.

We obtained the branching ratios from the FR ionization. As discussed in the main text, the peak e appears for both channels, and the peak a is only pronounced for the dissociative H_2 formation channel. The branching ratios of dissociation channel for the two electronic states can be also derived by the ratio of counts between two channels without the fitting procedure used in Figure 2 of the main text. The numbers of the electron events from the integration are shown in Supplemetary Table 6. The error range of branching ratios can be obtained by propagating the error of the counts of peak e or peak a. By comparing the results induced by TI and FR, we can claim that the obtained branching ratios are solely determined by the non-adiabatic coupling dynamics between the ground and excited cationic states.

88 TW/cm^2	peak e (Branching ratio)	peak a (Branching ratio)
Parent	$N_{e\mathrm{P}}$=307 ($N_{e\mathrm{P}}/N_e$ = 20(∓5)%)	$N_{a\mathrm{P}}$=29 ($N_{a\mathrm{P}}/N_a$ = 11(±10)%)
Dissociation	$N_{e\mathrm{D}}$=1246 ($N_{e\mathrm{D}}/N_e$ = 80(±5)%)	$N_{a\mathrm{D}}$=234 ($N_{a\mathrm{D}}/N_a$ = 89(∓10)%)
Total	N_e=$N_{e\mathrm{P}}$ + $N_{e\mathrm{D}}$=1553	N_a=$N_{e\mathrm{P}}$ + $N_{a\mathrm{D}}$=263

82 TW/cm^2	peak e (Branching ratio)	peak a (Branching ratio)
Parent	$N_{e\mathrm{P}}$=5564 ($N_{e\mathrm{P}}/N_e$ = 28(∓4)%)	$N_{a\mathrm{P}}$=764 ($N_{a\mathrm{P}}/N_a$ = 19(±8)%)
Dissociation	$N_{e\mathrm{D}}$=14337 ($N_{e\mathrm{D}}/N_e$ = 72(±4)%)	$N_{a\mathrm{D}}$=3241 ($N_{a\mathrm{D}}/N_a$ = 81(∓8)%)
Total	N_e=$N_{e\mathrm{P}}$ + $N_{e\mathrm{D}}$=19901	N_a=$N_{e\mathrm{P}}$ + $N_{a\mathrm{D}}$=4005

Supplementary Table 6: Branching ratios of H_2 formation and parent ion channel for two electronic states from Freeman resonance ionization. $N_{e\mathrm{P}}$ and $N_{a\mathrm{P}}$ stand for the total electron counts of peak e and peak a in the parent ion channel, $N_{e\mathrm{D}}$ and $N_{a\mathrm{D}}$ stand for the total electron counts of peak e and peak a in the H_2 formation channel, and N_e (or N_a) are the total counts of peak e (or a) measured in both two channels. The formula used to derive the branching ratios of H_2 formation channel and parent ion channel is shown in the bracket.

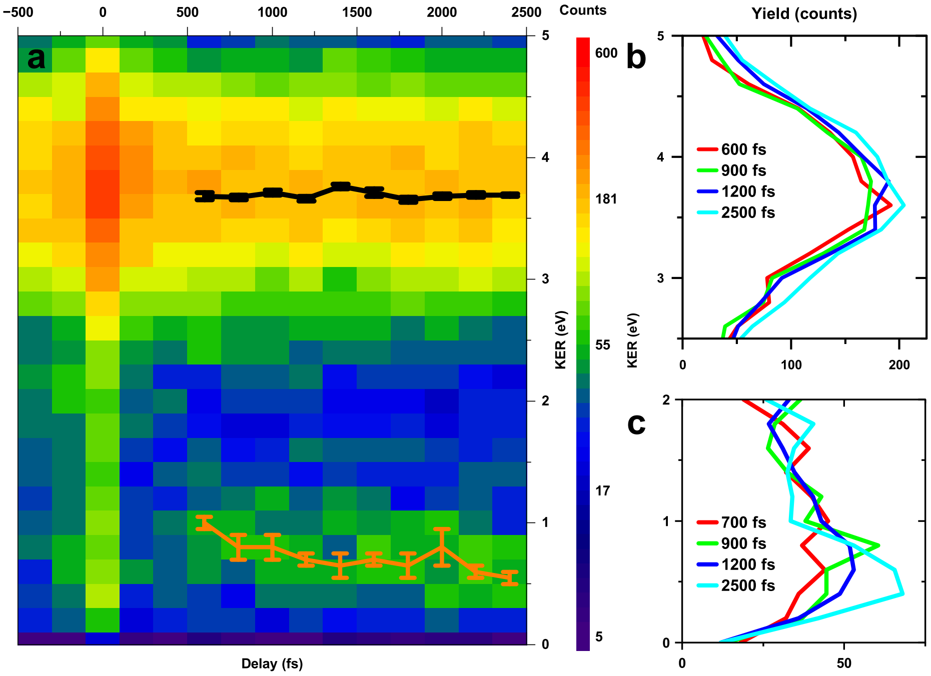

Supplementary Figure 10: Delay time-dependent yields and kinetic energy release (KER) of the two-body Coulomb explosion channel $C_2H_6^{2+} \to C_2H_4^+ + H_2^+$. a A plot of the KER vs pump-probe delay for this specific channel. **b, c** The distributions of KER and ion-pair yields at different time delays.

Supplementary note 7: Time-Dependent yield and KER of different channels

In our time-resolved measurement, the pump pulse triggers the neutral H_2 formation channel, where $C_2H_6^+$ dissociates into $C_2H_4^+$ and H_2. The probe pulse is used to monitor the formation dynamics by ionizing the transient $C_2H_4^+$, resulting in the formation of $C_2H_4^{2+}$ ($C_2H_4^+ + H_2 \to C_2H_4^{2+} + H_2$ (1)). Additionally, the probe pulse can also ionize H_2 to H_2^+, allowing the monitoring of the H_2 formation dynamics by detecting the delay time-dependent dynamics of the ion pair ($C_2H_4^+ + H_2^+$). Using the ion-ion coincidence technique with COLTRIMS, we obtained the delay time-dependent yield and kinetic energy release (KER) of the two-body Coulomb explosion channel ($C_2H_4^+ + H_2 \to C_2H_4^+ + H_2^+$ (2)). The results show a strong band with a KER of 3.8 eV that exhibits no delay time dependence (Supplementary Figure 10b), indicating that it originates from the direct Coulomb explosion of $C_2H_6^{2+}$ [7]. This pathway does not reflect the neutral H_2 formation dynamics (Supplementary Figure 10). Interestingly, a weak band appears after a time delay of approximately 700 fs, showing strong delay-time dependence. The KER of this band

decreases from 1.0 eV to 0.5 eV, and at the same time (Supplementary Figure 10c), the yield of this band clearly increases for time delays larger than 1000 fs. These features of the band can reflect the neutral H_2 formation dynamics. The appearance time of this band (approximately 700 fs) corresponds to the moment when the wave-packet starts to arrive at the CI and the H_2 formation channel becomes active, consistent with the results presented in the main text for channel (1). Due to the large-scale motion of the hydrogen atom during the non-adiabatic dynamics along the cation, the KER of this band is smaller compared to the direct Coulomb explosion channel from the dication. As the delay time increases, the distance between $C_2H_4^+$ and H_2 groups becomes larger, resulting in an even smaller KER (Supplementary Figure 10c). The neutral H_2 formation dynamics underlying the time-dependent features of the $(C_2H_4^+ + H_2^+)$ channel are overall consistent with channel (1). However, the yield of channel (2) is much smaller than channel (1), so the main focus in the main text is on channel (1).

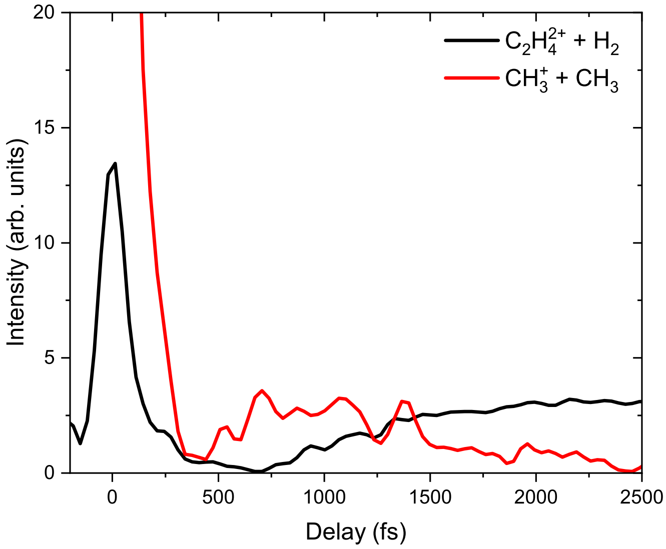

Supplementary Figure 11: The delay time-dependent yields of two different channels. Their yields exhibit opposite trend as increasing the time delay. Two curves are normalized to their minimum values.

At the early time before the wave-packet arrives to the CI, we observed another channel $C_2H_6^+ \rightarrow CH_3^+ + CH_3$ (3). This channel shows opposite time-dependent dynamics compared to channel (1) $(C_2H_4^{2+} + H_2)$, as shown in Supplementary Figure 11. Channel (3) originates from the dissociation of higher excited states of the cation. It exhibits a clear enhancement starting from 500 fs and starts to decrease after 1300 fs. Furthermore, the increasing counts of channel (1) and the decreasing counts of channel (3) are at the same level, suggesting that the excitation of $C_2H_6^+$ is an alternative pathway before the wave-packet arrives at the CI. After passing through the CI, the ionization of $C_2H_4^+$ becomes more significant relative to channel (3). This result indicates that the initial wave-packet, after ionization, can be promoted to higher excited states induced by the probe pulse before the $C_2H_4^+$ is reformed.

† Electronic address: zheng.li@pku.edu.cn
‡ Electronic address: xlhao@sxu.edu.cn
§ Electronic address: ccwang@jlu.edu.cn
¶ Electronic address: dajund@jlu.edu.cn

[1] Werner, H. *et al.* MOLPRO, version 2012.1, a package of ab initio programs (2012).
[2] Radziuk, D. & Möhwald, H. Ultrasonically treated liquid interfaces for progress in cleaning and separation processes. *Phys. Chem. Chem. Phys.* **18**, 21–46 (2016).
[3] Kotur, M., Weinacht, T. C., Zhou, C. & Matsika, S. Strong-field molecular ionization from multiple orbitals. *Phys. Rev. X* **1**, 021010 (2011).
[4] Krečinić, F. *et al.* Multiple-orbital effects in laser-induced electron diffraction of aligned molecules. *Phys. Rev. A* **98**, 041401 (2018).
[5] Zhang, J. *et al.* Channel-resolved multiorbital double ionization of molecular Cl_2 in an intense femtosecond laser field. *Phys. Rev. A* **98**, 043402 (2018).

[6] Yan, T.-M., Popruzhenko, S. V., Vrakking, M. J. J. & Bauer, D. Low-energy structures in strong field ionization revealed by quantum orbits. *Phys. Rev. Lett.* **105**, 253002 (2010).

[7] Kanya, R. *et al.* Hydrogen scrambling in ethane induced by intense laser fields: Statistical analysis of coincidence events. *The Journal of Chemical Physics* **136**, 204309 (2012).
